# Mood color choice helps to predict response to hypnotherapy in patients with irritable bowel syndrome

**DOI:** 10.1186/1472-6882-10-75

**Published:** 2010-12-07

**Authors:** Helen R Carruthers, Julie Morris, Nicholas Tarrier, Peter J Whorwell

**Affiliations:** 1 Department of Medicine, University of Manchester, Manchester, UK; 2 Department of Medical Statistics, Wythenshawe Hospital, Manchester, UK; 3 Division of Clinical Psychology, School of Psychological Sciences, University of Manchester, Manchester, UK

## Abstract

**Background:**

Approximately two thirds of patients with irritable bowel syndrome (IBS) respond well to hypnotherapy. However, it is time consuming as well as expensive to provide and therefore a way of predicting outcome would be extremely useful. The use of imagery and color form an integral part of the hypnotherapeutic process and we have hypothesised that investigating color and how it relates to mood might help to predict response to treatment. In order to undertake this study we have previously developed and validated a method of presenting colors to individuals for research purposes called the Manchester Color Wheel (MCW). Using this instrument we have been able to classify colors into positive, neutral and negative shades and this study aimed to assess their predictive role in hypnotherapy.

**Methods:**

156 consecutive IBS patients (aged 14-74, mean 42.0 years, 127 (81%) females, 29 (19%) males) were studied. Before treatment, each patient was asked to relate their mood to a color on the MCW as well as completing the IBS Symptom Severity Score, the Hospital Anxiety and Depression (HAD) Scale, the Non-colonic Symptom Scale, the Quality of Life Scale and the Tellegen Absorption Scale (TAS) which is a measure of hypnotisability. Following hypnotherapy all these measures were repeated with the exception of the TAS.

**Results:**

For patients with a positive mood color the odds of responding to hypnotherapy were nine times higher than that of those choosing either a neutral or negative color or no color at all (odds ratio: 8.889; p = 0.042). Furthermore, a high TAS score and the presence of HAD anxiety also had good predictive value (odds ratio: 4.024; p = 0.092, 3.917; p < 0.001 respectively) with these markers and a positive mood color being independent of each other. In addition, these factors could be combined to give an even stronger prediction of outcome. Twice as many responders (63, 77.8%) had a positive mood color or were anxious or had a high TAS score compared with 32 (42.7%) without these factors (p < 0.001).

**Conclusion:**

A positive mood color, especially when combined with HAD anxiety and a high TAS score, predict a good response to hypnotherapy.

## Background

**Figure 1:**
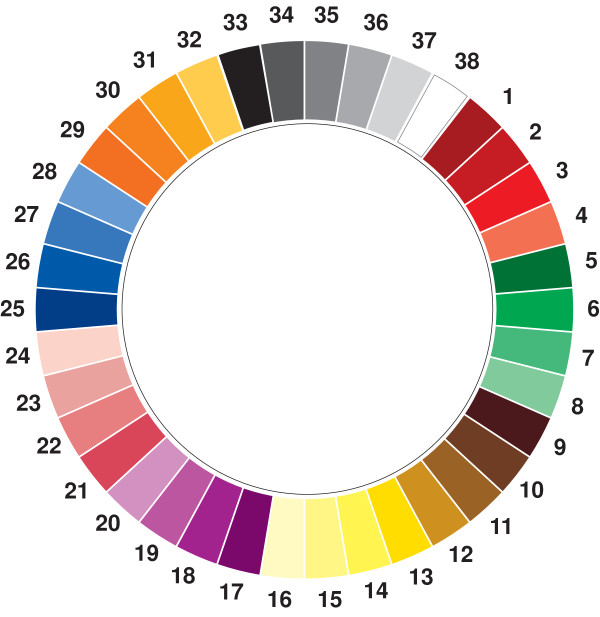
**The Manchester Color Wheel**.

We have previously shown that patients with irritable bowel syndrome (IBS) are more likely to respond to hypnotherapy (HT) treatment if they have an image of their illness and especially if that image is in color [1]. This led us to speculate that how a patient relates their illness and their mood to color might also help to predict outcome to treatment and it was the purpose of this study to test this hypothesis. In the absence of a simple way of presenting a series of colors to a patient for choice it was necessary to create a suitable instrument (Figure 1) which we called the Manchester Color Wheel (MCW) [2]. The development and validation of the MCW is described elsewhere and using this instrument it emerged that the positive, neutral and negative attributions of color appeared to be much more meaningful in relation to mood than an individual color itself. This was because different shades of the same color could have completely different connotations, for instance, pale blue was strongly positive whereas dark blue was negative. It was concluded that for quantitative research on color it may be more useful to relate findings to whether color choice is collectively positive or negative rather than concentrating on a particular color [2].

Data on color perception and mood suggests that black, brown and grey are consistently associated with negative emotions [3,4] and it has been shown that patients suffering from depression appear to have impaired color sensitivity [5] and view life monochromatically [6]. It has also been shown that depressed subjects are more likely to choose brown and black to describe their current mood whereas non-depressed individuals are more likely to choose colors which reflect their color preference [7]. Similarly, we have shown, using the MCW, that negative colors are more likely to be chosen by depressed subjects and that in most cases the higher the depression score the more likely they are to choose such a color. In addition we have found that up to 79% of people with an affective disorder equate their mood with a color compared to only 39% of healthy controls [2]. Thus, if an individual has something that affects their mood this makes them more likely to attribute a color to their state of mind.

**Figure 2:**
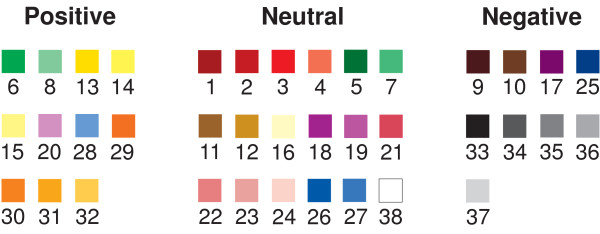
**Classification of colors from the MCW into positive, neutral and negative shades **[2].

This study aimed to assess whether hypnotherapeutic outcome could be predicted by relating mood to a positive, neutral or negative color (Figure 2) as well as seeking an association, if any, with symptom severity, hypnotisability or anxiety and depression.

## Methods

156 consecutive patients (aged 14-74, mean 42.0 years, 127 (81%) females, 29 (19%) males) attending the Hypnotherapy Unit at Wythenshawe Hospital who fulfilled the Rome II criteria [8] for IBS were studied. Non-colonic symptoms are a prominent and intrusive feature of IBS [9] and were recorded in addition to all the classic symptoms of IBS. Patients suffering from any significant psychiatric disease or any other serious concomitant disease such as diabetes or heart disease were also excluded from the study as were individuals with color blindness and pregnant women.

Prior to consultation with the hypnotherapist, using the MCW [2], each patient was asked, *"With regard to your day-to-day mood over the last few months, do you associate it with a particular color? If so, which color?" *In addition, the validated Symptom Severity Questionnaire [10] was also completed, with a reduction in score of greater than 50 points being considered as clinically significant. The Hospital Anxiety and Depression (HAD) Scale [11] was also administered with a score of 10 or more for either anxiety or depression being regarded as abnormal. The Non-colonic Symptom Scale [12], and Quality of Life Scale [12] were then completed as well as the Tellegen Absorption Scale (TAS) [13], which is used to assess hypnotisability in terms of high, medium or low. The patients then attended for 12 sessions of 'gut-focused' HT [14], over a three-month period, usually at weekly intervals. They were then asked to evaluate the change in their symptoms, in percentage terms compared with how they were before the start of therapy. A responder was defined as an individual who experienced a 50% or more reduction in their overall symptoms of their IBS following treatment. Patients also repeated the IBS Symptom Severity Questionnaire, the HAD Scale, the Non-colonic Symptom Scale, and Quality of Life Scale and were again asked, *"With regard to your day-to-day mood over the last few months, do you associate it with a particular color? If so, which color?"*

The main objective of this study was to ascertain whether color choice could predict a good or poor outcome for patients undergoing HT for their IBS. In order to achieve this, the response rate was assessed in relation to their positive, neutral and negative color choices as well as those who chose no color at all ('none'). The data were analysed on an intention-to-treat basis in order to establish whether patients who failed to start or complete treatment had any particular characteristics with regard to their color choice.

### Statistical analysis

The statistical package SPSS 15 was used for analysing the data. Parametric tests were used for the Normally distributed data. The distribution of data was assessed by calculating skewness and kurtosis statistics. The Pearson Chi-squared test was used to assess relationships between categorical variables and the Fisher's Exact test where the volunteer numbers were small. Linear-by-linear associations were used to assess the linear trend across the three classifications of hypnotic ability of the TAS. Analysis of differences between multiple groups was carried out using one-way ANOVA, with Scheffé's [15] multiple comparison Post-hoc test. Logistic regression was carried out to assess whether mood color, TAS score and HAD score had an independent relationship with response to treatment or whether one of these factors was acting as a proxy for another. It should be noted that where patient numbers were relatively small, the statistical analysis needs to be regarded as exploratory rather than definitive. The results did show a firm trend and therefore the application of a Bonferonni correction was thought to be unnecessary.

Ethical approval was sought and obtained from South Manchester Research Ethics Committee and all subjects gave written consent before participating.

## Results

Of the 156 patients included in this study 95 (60.9%) responded to treatment, 41 (26.3%) were non-responders and 20 (12.8%) failed to start or complete treatment. With respect to the symptom severity score, responders exhibited a mean reduction of 146.

### Response to treatment

The color choice of patients pre-HT is shown in Table 1. As can be seen, 36.8% of responders (p = 0.003, Fisher's Exact test) associated a positive or neutral color with their mood as opposed to 14.7% of non-responders and non-finishers. In contrast, 85.2% of non-responders and non-finishers associated a negative color or no color at all with their mood compared with 63.2% of the responder group. When comparing positive color choice with neutral, negative and 'none' combined the difference was also statistically significant (p = 0.016, Fisher's Exact test).

**Table 1:** Response to hypnotherapy and mood color choice (pre-HT)

	Positive No. (%)	Neutral No. (%)	Negative No. (%)	None No. (%)
Responders (n = 95)	12 (12.6%)	23 (24.2%)	20 (21.1%)	40 (42.1%)

Non-responders & non-finishers (n = 61)	1 (1.6%)	8 (13.1%)	19 (31.1%)	33 (54.1%)

Chi-square (3) = 10.344; p = 0.016

#### Anxiety and depression

The above results were then re-analysed to see whether there were any differences between mood color choice and response to HT when patients were divided into those suffering from anxiety and depression according to the HAD Scale as well as those who were neither anxious nor depressed. Analyses were carried out by comparing the positive color group with the neutral, negative and 'none' groups combined and the results are shown in Table 2. As can be seen, the main differences were that non-responders consistently failed to choose a positive color to describe their mood irrespective of whether they were HAD normal, anxious or depressed. In addition, depressed individuals also failed to choose a positive color to describe their mood and were more likely to choose a negative color irrespective of whether they responded or not.

**Table 2:** Response to hypnotherapy and mood color choice in 'HAD normal', 'HAD anxious' and 'HAD depressed' patients (pre-HT)

	Positive No. (%)	Neutral No. (%)	Negative No. (%)	None No. (%)
**'HAD normal' (n = 52)**				

Responders (n = 26)	6 (23.1%)	6 (23.1%)	4 (15.4%)	10 (38.5%)
Non-responders & non-finishers (n = 26)	0 (0%)	6 (23.1%)	4 (15.4%)	16 (61.5%)

**'HAD anxious' (n = 67)**				

Responders (n = 52)	6 (11.5%)	14 (26.9%)	10 (19.2%)	22 (42.3%)
Non-responders & non-finishers (n = 15)	0 (0%)	1 (6.7%)	3 (20.0%)	11 (73.3%)

**'HAD depressed' (n = 35)**				

Responders (n = 17)	0 (0%)	3 (17.6%)	6 (35.3%)	8 (47.1%)
Non-responders & non-finishers (n = 18)	1 (5.6%)	1 (5.6%)	11 (61.1%)	5 (27.8%)

Fisher's Exact Test: Positive versus neutral, negative and none combined

'HAD normal' p = 0.023; 'HAD anxious' p = 0.33; 'HAD depressed' p = 1.0

### Associations of mood color with Hospital Anxiety and Depression (HAD) score, Tellegen Absorption Scale (TAS) score, symptom severity, non-colonic symptomatology and quality of life, pre-hypnotherapy (pre-HT)

#### Hospital Anxiety and Depression (HAD) score (pre-HT)

The actual mean scores for anxiety and depression were also related to pre-HT mood color choice groupings (positive, neutral and negative), irrespective of response to treatment, using ANOVA (Table 3). Multiple comparisons were carried out using the Scheffé Post-hoc test on the same data. As can be seen there was a significant association between anxiety (p = 0.036) and depression (p < 0.001) scores and color choice groupings. Patients who chose negative colors were more likely to have higher anxiety and depression scores. In contrast, patients who chose positive colors were more likely to have lower anxiety and depression scores. Post-hoc tests revealed a trend towards significance between the positive and negative groups (p = 0.91) for anxiety. With regard to depression, statistical differences were found between the positive and negative (p < 0.001) and neutral and negative (p = 0.001) groups.

**Table 3:** Association between pre-HT anxiety and depression score and pre-HT mood color choice

		*95% confidence interval*		
Mood color (n = 82)	Mean score	*Lower limit*	*Upper limit*	p value
**Anxiety**				
Positive (n = 13)	10.31	8.52	12.09	*ANOVA:* *f (2,79) = 3.476; p = 0.036*
Neutral (n = 31)	11.23	9.64	12.81	
Negative (n = 38)	13.45	11.86	15.04	

**Depression**				

Positive (n = 13)	4.62	2.83	6.40	*ANOVA:* *f (2,79) = 12.287; p < 0.001*
Neutral (n = 31)	5.87	4.82	6.92	
Negative (n = 38)	9.13	7.85	10.41	

*Out of a total of 156 patients, one patient failed to complete the HAD questionnaire satisfactorily and had to be omitted from the analysis and 73 patients chose no color at all*.

#### Tellegen Absorption Scale (TAS) score (pre-HT)

Patients scoring between 26-34 points on the TAS were classed as having a high hypnotic ability, between 16-25 medium and between 0-15 points low hypnotic ability. There was a significant association between the two mood color groups (positive versus neutral, negative and no color choice combined) and TAS scores in that those with a higher TAS score were more likely to choose positive colors compared with those with lower scores (Table 4). Two patients out of a total of 156 failed to complete the TAS questionnaire satisfactorily and had to be omitted from the analysis.

**Table 4:** Association between TAS score (hypnotic ability) and mood color choice

Mood color (n = 154)	TAS score			p value
	High	Medium	Low	
Positive (n = 13)	2 (16.7%)	8 (12.5%)	3 (3.8%)	*Linear trend: Chi-square (1) = 4.353; p = 0.037*
'Others' (n = 141)	10 (83.3%)	56 (87.5%)	75 (96.2%)	

#### Symptom Severity, Non-colonic Symptoms and Quality of Life (pre-HT)

Mood color was not significantly associated with symptom severity or the non-colonic symptoms. However, there was a significant association with quality of life. Table 5 describes the association with mood color in more detail showing that patients who chose negative colors were more likely to have a poorer quality of life (p = 0.008). Post-hoc tests revealed a significant difference between the positive and negative groups (p = 0.041) and the neutral and negative groups (p = 0.033).

**Table 5:** Association between pre-HT quality of life score and pre-HT mood color choice

Mood color (n = 82)	Mean score	*95% confidence interval*		p value
		*Lower limit*	*Upper limit*	
Positive (n = 13)	216.23	175.81	256.65	*ANOVA:* f (2,79) = 5.137; p = 0.008
Neutral (n = 31)	231.03	207.57	254.49	
Negative (n = 38)	283.58	251.75	315.41	

*Out of a total of 156 patients, one patient failed to complete the quality of life questionnaire satisfactorily and had to be omitted from the analysis and 73 patients chose no color at all*.

### Associations of mood color with Hospital Anxiety and Depression (HAD) score, Tellegen Absorption Scale (TAS) score, symptom severity, non-colonic symptomatology and quality of life, post-hypnotherapy (post-HT)

#### Hospital Anxiety and Depression (HAD) score (post-HT)

ANOVA was used to compare the post-HT values of anxiety and depression and the individual color choice groupings (positive, neutral and negative) with regard to post-HT mood color. Multiple comparisons were carried out using the Scheffé Post-hoc test on the same data.

There was a significant association between depression scores and the color choice with regard to mood color (p = 0.015) although not with anxiety (p = 0.27). Table 6 describes the association with depression in more detail showing that patients who chose negative colors were more likely to have higher depression scores. Post-hoc tests revealed that there was a statistically significant difference between the positive and negative groups (p = 0.023).

**Table 6:** Association between post-HT depression score and post-HT mood color choice

Mood color (n = 87)	Mean score	*95% confidence interval*		p value
		*Lower limit*	*Upper limit*	
Positive (n = 53)	3.53	2.53	4.53	*ANOVA:* *f (2,84) = 4.418; p = 0.015*
Neutral (n = 26)	4.96	3.49	6.44	
Negative (n = 8)	7.75	2.32	13.18	

*Out of a total of 156 patients, 20 were non-finishers and 49 patients chose no color at all*.

#### Symptom Severity, Non-colonic Symptoms and Quality of Life (post-HT)

There was a significant relationship between the post-HT symptom severity, the non-colonic and quality of life scores and mood color (p = 0.013, p = 0.024, p = 0.014 respectively). Table 7 describes these associations in more detail showing that patients who chose negative colors were more likely to have higher symptom severity scores, higher non-colonic scores and worse quality of life whereas patients who chose positive colors had lower symptom severity scores and better quality of life. Post-hoc tests revealed significant differences between the positive and negative groups for symptom severity, non-colonic score and quality of life (p = 0.018, p = 0.029, p = 0.020 respectively). Furthermore, there was also a significant difference between the neutral and negative groups with respect to the non-colonic symptom score (p = 0.042).

**Table 7:** Association between post-HT symptom severity, non-colonic and quality of life scores and post-HT mood color choice

Mood color (n = 87)	Mean score	*95% confidence interval*		p value
		*Lower limit*	*Upper limit*	
**Symptom severity**				

Positive (n = 53)	169.23	142.13	196.32	*ANOVA:* *f (2,84) = 4.591; p = 0.013*
Neutral (n = 26)	207.77	160.96	254.58	
Negative (n = 8)	290.88	153.95	427.80	

**Non-colonic**				

Positive (n = 53)	158.85	137.02	180.68	*ANOVA:* *f (2,84) = 3.879; p = 0.024;*
Neutral (n = 26)	158.04	122.97	193.11	
Negative (n = 8)	249.13	135.06	363.19	

**Quality of life**				

Positive (n = 53)	156.34	135.85	176.83	*ANOVA:* *f (2,84) = 4.528; p = 0.014*
Neutral (n = 26)	184.85	154.40	215.29	
Negative (n = 8)	241.13	148.90	333.35	

*Out of a total of 156 patients, 20 were non-finishers and 49 patients chose no color at all*.

### Predictive value of color choice

As already described, mood color had predictive value with regard to response to treatment (Table 1). It was considered that other factors such as anxiety, depression or absorption might also have an effect. Consequently a univariate analysis was undertaken to establish the effect, if any, of these variables. The results are shown in Table 8 and, as can be seen, TAS score and HAD anxiety score were significant predictors of response to HT treatment (p = 0.008 and p = 0.002 respectively).

**Table 8:** Univariate analysis relating TAS and HAD score to response to treatment

	Responders (n = 95) No. (%)	Non-responders & non-finishers (n = 59) No. (%)	p value
**TAS score**			

High	10 (10.5%)	2 (3.4%)	*Chi-square (2) = 9.698; p = 0.008*
Medium	46 (48.4%)	18 (30.5%)	
Low	39 (41.1%)	39 (66.1%)	

**HAD score**			

'HAD normal'	26 (27.4%)	26 (44.1%)	*Chi-square (2) = 12.742; p = 0.002*
'HAD anxious'	52 (54.7%)	15 (25.4%)	
'HAD depressed'	17 (17.9%)	18 (30.5%)	

Logistic regression was carried out in order to assess whether these factors, as well as mood color, had an independent relationship with response to treatment or whether one factor was acting as a proxy for another, in other words, these predictive factors were interrelated. Table 9 confirms that each of these factors had a significant independent relationship with response to treatment, with the TAS score showing less evidence of predictive power. For patients with a positive mood color the odds of responding to HT were nearly nine times higher than that of those choosing either a neutral or negative color or no color at all. Furthermore, for patients who were anxious or had a high TAS score, the odds of responding were approximately four times higher compared to the reference groups.

**Table 9:** Logistic regression assessing the relationship between response to treatment and pre-HT mood color, TAS and HAD scores respectively

Category	p-value	Odds ratio
Pre-HT mood color (positive color) *(reference group = neutral, negative or no color at all)*	0.042	8.889

TAS score (high score) *(reference group = medium or low score)*	0.092	4.024

HAD score (HAD anxious) *(reference group = 'HAD normal' or 'HAD depressed')*	<0.001	3.917

Further analysis was undertaken to establish the predictive value of having one of the markers described in Table 9, and a positive response to treatment. Twice as many responders (63, 77.8%) were either anxious or had a high TAS score or chose a positive color to describe their mood, compared with 32 (42.7%) without any of these features (Chi-square (1) = 20.161; p < 0.001). Furthermore, the presence of two or more of these markers guaranteed a response to treatment (p = 0.007, Fisher's Exact test).

## Discussion

This study has established that color choice helps to predict response to HT. For patients relating their mood to a positive mood color, the odds of responding to HT were nearly nine times higher than that of those choosing either a neutral or negative color or no color at all. HAD anxiety and a high TAS score were also found to be useful factors in determining a good response to treatment.

Our Unit has been providing HT for the treatment of IBS for over twenty years with approximately two thirds of patients responding to treatment [16]. Unfortunately, patients may require as many as twelve one hour sessions of therapy to secure a response and consequently this results in the treatment being relatively expensive to provide. It would therefore be extremely useful if there were a way of predicting those who would or would not respond to treatment. If a sufficiently robust way of singling out potential responders could be identified this would save the third of individuals who fail to respond, the disappointment of going through unsuccessful HT. This group could then be offered some alternative management strategy.

Imagery plays an important part in the hypnotherapeutic process and we have previously shown that patients, who have an image of their disorder, especially if it is in color, are more likely to respond to this particular form of treatment [1]. However, the predictive value of having an image is not exact and therefore additional measures are necessary. We therefore hypothesised that how a patient relates to color might have additional value in defining a responder. In a pilot study we assessed mood color, favourite color, drawn to color and color of illness and found that only mood color appeared to have potential for the purposes of this study.

In the present study the response rate to HT was 61% which is slightly lower than we have previously observed [16]. This is because we included patients who did not even start treatment following consultation with the hypnotherapist as we wanted to try and define the characteristics of patients who were clearly unsuited to this form of treatment from the outset. In addition to the clear cut relationship of a positive mood color, HAD anxiety and a high TAS score with a good hypnotherapeutic outcome, there were a number of other significant observations of interest. For instance before HT, even if responders did not choose a positive color, they significantly associated their mood with a neutral color as opposed to non-responders and non-finishers who chose negative colors or no color to describe their mood (p = 0.016). Furthermore, if a patient chose a positive color to describe their mood and had a normal HAD score they were even more likely to respond. Interestingly, 23% of 'HAD normal' and 12% of 'HAD anxious' responders chose a positive color to describe their mood compared with none of the 'HAD depressed' responders. In contrast, only one patient in the non-responder group chose a positive shade. Gender appeared to have little effect on any of the results.

We have previously shown that patients suffering from depression are very unlikely to choose a positive shade to describe their mood [2]. Consequently, it might be concluded that, as a positive shade is a predictor of a good outcome with HT, IBS patients exhibiting depression could be deemed as unsuitable for this form of treatment because they are likely to choose a negative color. However, this analysis has shown that the depressed patients who responded to HT had completely different characteristics to depressed non-responders. Firstly, fewer selected a negative color and this could be interpreted as either they are not so entrenched in their depression or that they differ from endogenously depressed patients in that they are depressed because of their illness, rather than just being depressed and this is easier to manage. Another characteristic was that they were more likely to choose a neutral color or no color at all and this could be interpreted as meaning that their depression was not so 'dark' indicating the ability to see a way out of their condition. Therefore, in depressed IBS patients being considered for HT, those choosing neutral colors or no color at all to describe their mood should certainly not be excluded from being given the opportunity to receive this form of treatment.

Patients with high TAS scores were more likely to choose positive shades in relation to mood colors. Conversely, those with medium or low scores more frequently selected a neutral or negative color or no color at all. This might be explained by the fact that the TAS questionnaire is, in some ways, a test of the imagination which is an important component of hypnotic ability. Therefore individuals scoring highly on such an instrument might be expected to more easily give answers in response to questions relating to color. Given that patients with depression seldom choose positive colors [2], it would be of interest to explore the prevalence of depression in individuals, irrespective of whether they have IBS, scoring highly on the TAS scale which appears to be strongly associated with a positive mood color choice. With regard to actual HAD scores, patients who chose a negative color were more likely to have higher anxiety and depression scores. Conversely, patients who chose a positive color to describe their mood were significantly more likely to have lower anxiety and depression scores. This result confirms our previous findings showing that negative colors were more likely to be chosen by depressed subjects and that in most cases the higher the depression score the more likely they are to choose such a color [2]. Overall the mean HAD scores fell substantially following HT and although some patients still fell into the category of depression their overall HAD scores had improved. Another notable point was that the number of patients choosing a positive shade to describe their mood increased from 13 to 53 patients following HT and the number of patients choosing a negative shade decreased from 38 to 8 patients following treatment. Mood color choice was also seen to be significantly associated with IBS symptom severity, non-colonic symptom score and quality of life following HT.

## Conclusion

Mood color choice, anxiety and a high TAS score are independent predictors of good outcome to HT which can be combined to strengthen their predictive value. It may be that in the future data such as these could be combined with our previous observations on imagery [1,17] to give an overall score with thresholds for predicting a poor, moderate or good response to HT.

## Abbreviations

IBS: Irritable Bowel Syndrome; HT: Hypnotherapy; MCW: Manchester Color Wheel; HAD: Hospital Anxiety and Depression Scale; TAS: Tellegen Absorption Scale

## Competing interests

The authors declare that they have no competing interests.

## Authors' contributions

HRC participated in the design of the study, collected and analysed all the data as well as drafting the manuscript. JM advised on the statistical analysis of the data and checked the accuracy of the final results. NT participated in the design of the study, acted as PhD supervisor to HRC and reviewed and critiqued the manuscript. PJW conceived the project and participated in its design as well as finalising the manuscript. All authors have read and approved the final manuscript.

## Pre-publication history

The pre-publication history for this paper can be accessed here:

http://www.biomedcentral.com/1472-6882/10/75/prepub
